# Predictors of unsuccessful tuberculosis treatment outcomes in children from a prospective cohort study in Pakistan

**DOI:** 10.7189/jogh.11.04011

**Published:** 2021-02-11

**Authors:** Meredith B Brooks, Amyn Malik, Salman Khan, Junaid F Ahmed, Sara Siddiqui, Maria Jaswal, Saniya Saleem, Farhana Amanullah, Mercedes C Becerra, Hamidah Hussain

**Affiliations:** 1 Department of Global Health and Social Medicine, Harvard Medical School, Boston, Massachusetts, USA; 2 Global Health Directorate, Indus Health Network, Karachi, Pakistan; 3 Yale Institute for Global Health, New Haven, Connecticut, USA; 4 Interactive Research and Development Global, Singapore; 5 The Indus Hospital, Karachi, Pakistan

## Abstract

**Background:**

Every year, about 239 000 children die from tuberculosis (TB), despite availability of highly effective regimens. Few studies have evaluated predictors for poor treatment outcomes in children treated for TB.

**Methods:**

We assessed predictors of unsuccessful TB treatment outcomes in a prospective cohort of children diagnosed by an intensified TB patient-finding intervention at four facilities in Pakistan between 2014 and 2016. A case of TB disease was determined through either bacteriologic confirmation of disease or a clinical diagnosis. To estimate characteristics predictive of experiencing an unsuccessful treatment outcome, we used a multi-level model with a modified Poisson approach, accounting for clustering at the facility level. We report estimated relative risks (RR) and 95% confidence intervals (CI).

**Results:**

During the study period, 1404 children less than 15 years old were initiated on treatment for drug-susceptible TB. In total, 709 (50.5%) were 0-4, 406 (28.9%) were 5-9 years, and 289 (20.6%) were 10-14 years old; 614 (43.7%) were female; and of the 1377 children assessed for malnourishment, 1161 (84.3%) were malnourished. A total of 1322 (94.2%) children experienced a successful treatment outcome, 14 (1.0%) children transferred out to a different facility, and 68 (4.8%) children experienced an unsuccessful treatment outcome: 14 (1.0%) died, 20 (1.4%) failed treatment, and 34 (2.4%) were lost to follow-up. After adjustment for age group, sex, and malnutrition status, we identified increased risk of unsuccessful treatment outcome in children presenting with fever (RR = 2.56, 95% CI = 1.02-6.44; *P* = 0.05) or an abdominal examination suggestive of TB disease (RR = 2.34, 95% CI = 1.20-4.58; *P* = 0.01), and a decreased risk in children who initiated treatment at a rural facility (RR = 0.05, 95% CI = 0.00-0.74; *P* = 0.03).

**Conclusions:**

More than 94% of children experienced successful treatment outcomes. We identified individual-, facility-, and clinical-factors predictive of experiencing unsuccessful treatment outcomes. Children with fevers and abdominal findings suggestive of TB disease should be tested for TB and followed closely throughout treatment to ensure necessary support for successful completion of treatment.

Tuberculosis (TB) is a preventable infectious disease, yet one million children less than 15 years of age fall sick with TB each year [1-3]. Worldwide, every year 239 000 children die from TB [4]. Non-specific symptoms, difficulty producing a sputum sample for bacteriologic confirmation of disease, and low sensitivity of diagnostic testing due to the paucibacillary nature of pediatric TB can lead to delays in diagnosis and initiation of life-saving treatment [5-7]. The Childhood TB Roadmap, with a goal of zero TB deaths in children, has highlighted the importance of identifying strategies to support children and their families to improve treatment completion percentages and prevent loss to follow-up [8]. Currently TB programs do not report child and adolescent TB outcomes to the World Health Organization. Treatment outcomes data are an essential component of the pediatric patient pathway and critical to formulate and implement contextual targeted strategies.

Among the subgroup of children who are diagnosed with TB disease and who are successfully initiated on appropriate TB treatment, there are several previously identified factors that may increase a child’s risk of experiencing an unsuccessful treatment outcome, including death or loss to follow-up. Children who are less than five years old [9-15], have HIV co-infection [10,11,13,16-19], have a low body weight [18,20], and are bacteriologically positive [9,19,21] have an increased risk of experiencing unsuccessful treatment outcomes. While individual-level predictors have been assessed for their predictive ability of treatment outcomes in several studies, there is limited evidence on the role of facility-level characteristics and disease presentation, such as examination findings for TB disease. These predictors may vary by setting and population.

Pakistan accounts for approximately 6% of the global TB burden and has a TB incidence of 263 per 100 000 population; children make up 14% of the total TB burden [3]. It is essential to understand local predictors, including individual-, clinical- and facility-based factors, for unsuccessful treatment outcomes to identify local strategies that can be implemented to better support children with TB and their families [22]. Here we aimed to identify predictors of unsuccessful TB treatment outcomes in children 0-14 years of age who were treated for drug-susceptible (DS-) TB in Pakistan.

## METHODS

### Study design and population

Between October 2014 and March 2016, an intensified TB patient-finding intervention was implemented in collaboration with the Provincial TB Control Program with the aim of increasing childhood TB detection and notification through systematic verbal screening of all people seeking health care at four participating health facilities in Jamshoro district, Sindh, Pakistan. More details about this intensified TB patient-finding intervention have been reported previously [23,24].

### Key procedures and measures

Community health workers administered verbal TB symptom screening questionnaires (cough, fever, night sweats, and weight loss), adapted from the World Health Organization and National TB Control Program’s pediatric TB screening tool [25,26], to the guardians of children (aged 0-14 years) who visited the general, pediatric, and chest out-patient departments at the four participating health facilities. At the time of screening verbal assent was taken from the child’s guardian; if the child was seven years or older, verbal assent was also taken from the child. The community health workers also asked about exposure to persons with TB disease in the last two years and directly recorded all responses into an electronic mobile data capture system that was built specifically for this project with a decision support system to reduce chances of data entry errors and allow for real-time data monitoring. Children were referred for further evaluation and diagnostic testing to a TB medical officer if they either (a) reported contact with someone with TB disease, (b) presented with a cough lasting two or more weeks, or (c) reported any two additional symptoms (fever lasting  ≥ 2 weeks, night sweats, or weight loss).

All children had a chest x-ray and a complete blood count/erythrocyte sedimentation rate, along with Xpert MTB/RIF testing if they were able to expectorate sputum. Ultrasound, computed tomography, and fine-needle aspiration or biopsy were performed based on clinical indication. A case of TB disease was determined through either bacteriologic confirmation of disease or a clinical diagnosis. Children who were clinically diagnosed were assumed to have drug-susceptible TB disease unless they reported a history of contact to a patient with drug-resistant TB. Children diagnosed with drug-susceptible TB disease were initiated on treatment per the Provincial TB Program Sindh guidelines [26] with a six-month drug-susceptible regimen of two months rifampin, isoniazid, pyrazinamide, and ethambutol followed by four months of rifampin and isoniazid. A robust monitoring and evaluation system was built into the study, with support from the Provincial TB Program Sindh, to ensure that, at each site, the appropriate diagnostic algorithm was followed, over-diagnosis was minimized, and linkage to treatment was ensured.

As part of routine care, children were followed throughout the duration of treatment until a treatment outcome was experienced, per standard definitions [26,27]. These standard definitions were used for all children who provided sputum samples for bacteriologic testing. For children unable to provide a sputum sample and who were diagnosed based on clinical examination, we defined treatment failure as a lack of response to 3–5 months of compliant (consistently taking medications based on self-report) TB treatment, as evidenced by a persistence of symptoms, weight loss or no weight gain, and/or no change or worsening of the chest x-ray. For this analysis, we defined two composite treatment outcome categories: successful (cure and treatment completion) or unsuccessful (death, treatment failure, or lost to follow-up). Children classified as transferred out to another facility were excluded from the analysis because treatment outcomes were not available for analysis.

Children’s demographic, clinical, diagnostic, and treatment-related characteristics were entered into the electronic data capture system. Demographic information included sex, age, weight, and the hospital where the child was enrolled for TB treatment. Weight-for-age percentile was also recorded at each visit, using the World Health Organization’s growth charts for children 0-2 years old and the US Centers for Disease Control and Prevention’s growth charts for children older than two years old.[28] We categorized a child as malnourished if his or her weight-for-age was at or below the fifth percentile. Children were categorized by age group, 0-4, 5-9, and 10-14 years old. Clinical characteristics included results of chest, abdominal, and lymph node examinations, chest x-ray results, symptoms, family history of TB, type of TB for the current diagnosis (pulmonary or extra-pulmonary), and the site if extra-pulmonary disease is present. Facility-based characteristics were also assessed, including whether the facility where the child initiated TB treatment served a rural or urban population, and whether the facility was a newly established TB treatment facility or not. Newly established TB treatment facilities were existing tertiary care hospitals that were not designated as a national TB reporting center prior to implementation of this project.

### Analysis

We report the frequency and percentage of baseline characteristics and treatment outcomes of children treated for TB in the four participating facilities. We used a multi-level model with a modified Poisson approach to estimate the relative risk (RR) and 95% confidence intervals (CI) using robust error variances [29] of the association of all characteristics with experiencing an unsuccessful treatment outcome. The model accounts for clustering at the facility level; random intercepts for each facility were produced. Covariates predictive of unsuccessful outcomes in univariable analysis with a *P* ≤ 0.20 were considered as candidates for inclusion in the final multivariable model. Covariates retained in the final model were those that predicted an unsuccessful treatment outcome (*P* ≤ 0.05), as well as age, gender, and malnourishment because of their established predictive ability with TB treatment outcomes. Missing data were less than 10%, so complete case analysis was used. All tests are two-sided with an alpha of 0.05. Analyses were complete in SAS version 9.4 (SAS Institute Inc., Cary, NC, USA).

### Ethical considerations

The Institutional Review Board (IRB) of Interactive Research and Development, Karachi, Pakistan, reviewed and approved the study protocol. The subsequent analysis of de-identifiable data was determined to be non-human subjects research by the IRB of Harvard Medical School.

## RESULTS

At the four participating hospitals, there were 1417 children less than 15 years of age diagnosed with drug-susceptible tuberculosis. Of these children, 1404 (99.1%) were linked to care and initiated TB treatment. The majority were male (790; 56.3%), half (708; 50.5%) were 0-4 years old, 406 (28.9%) were 5-9 years old, and 289 (20.6%) were 10-14 years old (**Table 1**). A total of 116 (84.3%) of children presented as malnourished. A majority of children had symptoms, with 1186 (84.5%), 1057 (75.6%), and 1239 (93.0%) reporting fever, weight loss, and cough, respectively. Chest examinations and x-rays were suggestive of TB disease in 1153 (84.0%) children, while abdominal examinations and lymph node examinations were suggestive of TB disease in 133 (10.3%) and 139 (10.8%) children, respectively. Only 42 (3.0%) children were bacteriologically positive at baseline. A total of 246 (17.5%) of children had extra-pulmonary involvement, with lymph node (126, 51.6%) and abdominal (103, 42.2%) involvement being most frequent. A total of 1080 (92.0%) of children had a parent with TB disease. Seventy-eight (5.6%) children were diagnosed and initiated on treatment at a new TB treatment facility, and 258 (18.4%) were diagnosed and initiated on treatment at a facility that serves a rural population.

**Table 1:** Baseline characteristics of 1404 children treated for DS-TB

Characteristic		Total children		Children experiencing successful treatment outcomes		Children experiencing unsuccessful treatment outcomes	
N*	**n (%)**	N*	**n (%)**	N*	**n (%)**
**Demographics**							
Female		1404	614 (43.7)	1322	575 (43.5)	68	31 (45.6)
Age group (years)	0-4	1404	709 (50.5)	1322	670 (50.7)	68	33 (48.5)
	5-9		406 (28.9)		381 (28.8)		20 (29.4)
	10-14		289 (20.6)		271 (20.5)		15 (22.1)
**Symptoms and clinical presentation**							
Weight-for-age <5^th^ percentile		1377	1161 (84.3)	1296	1085 (83.7)	67	62 (92.5)
Any cough present		1333	1239 (93.0)	1253	1165 (93.0)	66	60 (90.9)
Cough duration	No cough	1207	93 (7.7)	1053	93 (8.8)	48	2 (4.2)
	<2 weeks		96 (8.0)		313 (29.7)		16 (33.3)
	2-3 weeks		335 (27.7)		647 (61.4)		30 (62.5)
	>3 weeks		683 (56.6)		0 (0)		0 (0)
Fever present		1403	1186 (84.5)	1321	1110 (84.0)	68	62 (91.2)
Weight loss present		1399	1057 (75.6)	1320	992 (75.2)	66	53 (80.3)
Chest x-ray suggestive of TB		1372	1153 (84.0)	1291	1087 (84.2)	67	54 (80.6)
Chest examination suggestive of TB		1286	1111 (86.4)	1207	1045 (86.6)	65	53 (81.5)
Abdominal examination suggestive of TB		1286	133 (10.3)	1205	115 (9.5)	65	14 (21.5)
Lymph node examination suggestive of TB		1286	139 (10.8)	1199	119 (9.9)	63	10 (15.9)
BCG scars		1235	789 (63.9)	1159	739 (63.7)	63	42 (66.7)
Type of TB in child	Pulmonary	1404	1158 (82.5)	1322	1092 (82.6)	68	53 (77.9)
	Extra-pulmonary		246 (17.5)		230 (17.4)		15 (22.1)
Extra-pulmonary site	Abdomen	244	103 (42.2)	228	96 (42.1)	15	6 (40.0)
	Bone		2 (0.8)		2 (0.9)		0 (0)
	CNS		5 (2.1)		3 (1.3)		2 (13.3)
	Lymph node		126 (51.6)		122 (53.5)		4 (26.7)
	Pleural		6 (2.5)		4 (1.8)		2 (13.3)
	Other		2 (0.8)		1 (0.4)		1 (6.7)
**Family history of TB**							
Family history of TB		1404	1177 (83.8)	1322	1110 (84.0)	68	54 (79.4)
Family member with TB	Parent	1174	1080 (92.0)	1107	1017 (91.9)	54	52 (96.3)
	Sibling		25 (2.1)		23 (2.1)		2 (3.7)
	Grandparent		7 (0.6)		7 (0.6)		0 (0)
	Other		62 (5.3)		60 (5.4)		0 (0)
Family member with TB was sputum positive		1144	1069 (93.4)	1079	1007 (93.3)	53	51 (96.2)
Type of facility							
New TB treatment facility		1404	78 (5.6)	1322	61 (4.6)	68	14 (20.6)
Rural facility		1404	258 (18.4)	1322	257 (19.4)	68	1 (1.5)
**Treatment outcomes**							
Successful		1404	1322 (94.2)				
Cure		1322	11 (0.8)				
Treatment completed		1322	1311 (93.4)				
Unsuccessful		1404	68 (4.8)				
Died		68	14 (1.0)				
Treatment failure		68	20 (1.4)				
Loss to follow-up		68	34 (2.4)				
Transfer out		1404	14 (1.0)				

BCG – Bacille Calmette-Guerin vaccine, CNS – central nervous system, TB – tuberculosis

*N represents the number of children for which each variable have data reported; the remainder are missing. Thus, these values are the denominators for the percentages calculated in the subsequent columns.

Most children (1,322, 94.2%) experienced a successful treatment outcome (**Table 1**). Sixty-eight (4.8%) experienced unsuccessful treatment outcomes, including 14 (1.0%) deaths, 20 (1.4%) in whom treatment failed, and 34 (2.4%) who were lost to follow-up. An additional 14 (1.0%) children were transferred out to another facility. Because the treatment outcomes of the 14 children who were transferred out were not available for review, they were excluded from the primary analysis. Of children treated in each individual facility, the single rural facility had only one (0.4%) child experience an unsuccessful treatment outcome, the new TB treatment facility had 14 (18.7%) children experience an unsuccessful outcome, and the other two urban facilities had 20 (4.5%) and 33 (5.4%) children experience unsuccessful treatment outcome. Within the single new TB treatment facility, 9 (12.0%) of the children treated there were lost to follow-up, as compared to 11 (2.4%), 14 (2.3), and 0 (0%) children treated at the other facilities (the latter being the rural facility).

In univariable analysis, we identified that an abdominal examination suggestive of TB disease (RR = 2.42; 95% CI = 1.23-4.76) predicted experiencing unsuccessful treatment outcomes, while cough (RR = 0.30; 95% CI = 0.11, 0.81), a chest examination suggestive of TB (RR = 0.51; 95% CI = 0.26, 0.97), and treatment in a rural TB facility (RR = 0.05; 95% CI = 0.00-0.67) reduced the risk of experiencing unsuccessful treatment outcomes (**Table 2**). In multivariable analysis, when controlling for age group, sex, and malnutrition status, we identified an increased risk of an unsuccessful treatment outcome in children presenting with fever (RR = 2.56; 95% CI = 1.02-6.44) and children with abdominal examinations suggestive of TB disease (RR = 2.34; 95% CI = 1.20-4.58), and a reduced risk of an unsuccessful treatment outcome in children treated in a rural facility (RR = 0.05; 95% CI = 0.00-0.74). Of the children who experienced an unsuccessful treatment outcome, 62 (91.2%) had a fever. Of the 14 children with abdominal examinations suggestive of TB disease who experienced unsuccessful treatment outcome, 8 (57%) had extrapulmonary TB disease, of which 6 (75%) had abdominal TB.

**Table 2:** Univariable and multivariable analyses of child characteristics and unsuccessful treatment outcomes

Variable		Univariable RR (95% CI)	*P*-value	Multivariable RR (95% CI)	*P*-value
Female		1.07 (0.66, 1.73)	0.77	1.11 (0.68, 1.82)	0.68
Age group (years)	0-4	0.94 (0.51, 1.74)	0.85	0.92 (0.49, 1.70)	0.78
	5-9	1.02 (0.52, 1.99)	0.95	0.86 (0.43, 1.74)	0.68
	10-14	REF	REF	REF	REF
Weight-for-age <5^th^ percentile		0.73 (0.28, 1.87)	0.51	0.60 (0.21, 1.72)	0.34
Any cough present		0.30 (0.11, 0.81)	0.02		
Fever present		2.27 (0.97, 5.29)	0.06	2.56 (1.02, 6.44)	0.05
Weight loss present		1.75 (0.95, 3.25)	0.08		
Chest x-ray suggestive of TB		0.56 (0.28, 1.09)	0.09		
Chest examination suggestive of TB		0.51 (0.26, 0.97)	0.04		
Abdominal examination suggestive of TB		2.42 (1.23, 4.76)	0.01	2.34 (1.20, 4.58)	0.01
Lymph node examination suggestive of TB		1.82 (0.87, 3.82)	0.11		
BCG scars		1.03 (0.51, 2.11)	0.93		
Family history of TB		0.55 (0.28, 1.08)	0.08		
Type of TB in child (extrapulmonary vs pulmonary [reference])		1.57 (0.86, 2.89)	0.15		
New TB treatment facility		7.19 (0.48, 107.14)	0.15		
Rural facility		0.05 (0.00, 0.67)	0.02	0.05 (0.00, 0.74)	0.03

BCG – Bacille Calmette-Guerin vaccine, TB – tuberculosis, CI – confidence interval, RR – relative risk

**Figure 1:**
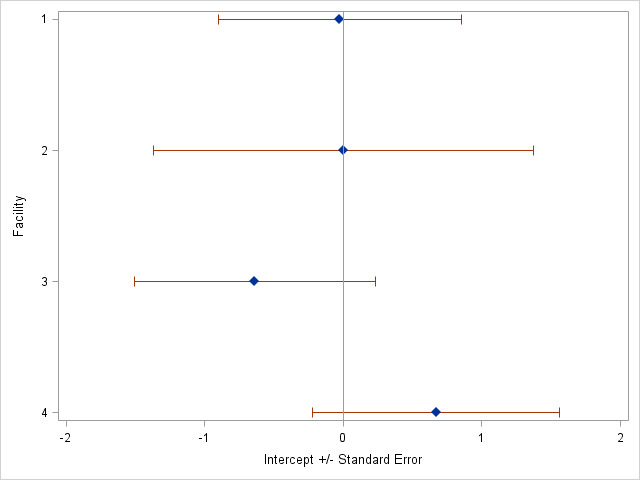
Random intercepts for facilities from multivariable analysis.

Additionally, we observe that the intercepts for each treatment facility vary, meaning each facility has different overall starting risks of the outcome (**Figure 1**). We observe that while the new TB treatment facility (Facility 4) had the highest intercept, and thus the highest starting risk of all the facilities, its standard error overlapped with the risks of the other treatment facilities.

## DISCUSSION

Overall, children treated for DS-TB at these four facilities in Pakistan experienced a very low percentage of unsuccessful treatment outcomes. Half of those unsuccessful outcomes were loss to follow-up and less than a quarter were deaths. These percentages are considerably lower than the majority of reports in the literature which, since 2016, range from 4.1% in The Netherlands [15] to 39.0% in Mauritania [12]. Two other recent reports of DS-TB treatment outcomes of children in Pakistan found that 4.9% [21] and 14.6% [9] experienced unsuccessful outcomes. Here, we likely observed low percentages of unsuccessful treatment outcomes due to the active, child-centered screening implemented at the facilities, routine referrals for TB examination when symptoms were present, and active follow up of children by study staff. This process may have identified children earlier in their disease course than usual, leading to an earlier diagnosis and more prompt initiation of life-saving treatment.

Additionally, we identified great variability by facility in loss to follow-up percentages, with the new TB treatment facility accounting for more than four times the loss to follow-up experienced at each of the other facilities. This suggests that more established TB treatment facilities may have had adequate systems in place for retaining patients and supporting them throughout treatment, whereas newer TB treatment facilities might need to focus more on training their doctors and staff and to incorporate patient centered care, including counseling services, to be able to accompany patients throughout the course of their treatment.

Although in this cohort, age, gender, and malnutrition were not identified to be predictors of unsuccessful treatment outcomes, we adjusted for these characteristics because previous reports have identified them to be predictors for poor tuberculosis treatment outcomes in children [9-20]. Those reports may have identified these factors as predictors of poor outcomes because the majority were set within programs relying only on passive TB finding, which is characterized by delayed diagnoses due to nonspecific symptoms and difficulty diagnosing young children who cannot produce a sputum specimen. The present study was an intensified TB patient-finding intervention [23,24], which was designed to identify these children earlier in their clinical disease course and initiate treatment more rapidly. Additionally, although bacteriological positivity [9,19,21] and HIV status [10,11,13,16-19] were identified in previous studies to increase the risk of a child experiencing an unsuccessful treatment outcome, we did not adjust for these variables in this analysis. Many of the children could not produce a sputum sample so bacteriologic positivity could not be assessed in the majority of children in this data set; only 3% were bacteriologically positive at baseline. Adjusting would have led to dropping observations from the primary analysis, leaving a very small sample size. Additionally, the prevalence of HIV is very low in this area, so much so that HIV is not routinely tested for in this population so data are not available. We do not anticipate that the exclusion of these variables would impact our final model and results.

Both fever and an abnormal abdominal examination were predictors of unsuccessful treatment outcomes. These children may have had relatively advanced disease that had been previously missed, thereby delaying access to appropriate care. Notably, fever at presentation was observed in more than 90% of the children who experienced an unsuccessful treatment outcome. Additionally, the majority of children with abdominal examinations suggestive of TB disease who experienced an unsuccessful treatment outcome were diagnosed with extrapulmonary TB disease, three-quarters of which were abdominal TB, while one child had pleural TB, one child had central nervous system TB, and the remaining children had pulmonary TB. For those children with abdominal TB, initial symptoms are often non-specific which can lead to a delay in diagnosis and, because microbiological confirmation of disease is difficult, diagnosis typically relies on clinical suspicion, imaging, histopathological findings, or response to treatment.[30] One study identified that abdominal pain, fever, and weight loss are the most common presenting symptoms of abdominal TB and that there is a median delay of diagnosis of four months [31].

We also identified that initiating treatment at a rural facility was protective against experiencing unsuccessful outcomes, even after adjusting for clustering by facility. The single rural facility had only 0.4% of children experience an unsuccessful treatment outcome, compared to 6% in the urban facilities. It is possible that sicker children were more likely to be referred to urban facilities for care; these facilities are larger hospitals, which offer a more broad range of diagnostics and care. If this were the case then the healthier children, at lower risk of poor outcomes, were more likely to be treated at the rural facilities. These findings are consistent with another study that found high percentages of successful TB treatment outcomes in children from Sindh, Pakistan, where a large proportion of the children were residents of a rural district [21].

The single new TB treatment facility had a higher proportion of unsuccessful outcomes (18.7%) compared to the other established TB treatment facilities (4.1%). While adjusting for clustering by facility should account for differential patient populations attending each facility, we still identified a large gap likely due to a lack of established processes. This large difference in outcomes underscores the need to increase staff training, and set procedures for logistic, diagnostic, and treatment support.

One limitation of this study is that we were unable to assess other known predictors for poor outcomes, such as comorbidities or social factors. However, comorbidities like diabetes, hepatitis, HIV, and chronic lung diseases are relatively low in this population. Socioeconomic status and education level of the parents are not routinely collected in the child’s medical chart. We recognize that such comorbidities and social factors may impact the child’s health or ability to complete treatment. Additionally, although we had a large sample size, only 68 children experienced an unsuccessful treatment outcome, potentially under-powering our study to identify other important predictors. Our study also had several strengths, including the prospective design – which enabled the direct estimation of relative risks – the large sample size of children, and the low levels of missing data.

## CONCLUSION

Our results can inform efforts to improve treatment outcomes of children with TB in Pakistan. Fever and abnormal abdominal examinations may indicate a relatively more advanced disease state in children, which can lead to an unsuccessful treatment outcome. Integration of pediatric TB symptom screening at all levels of health care, including general clinics, nutrition clinics, immunization centers, and chest disease clinics, with appropriate referrals and family support will reduce late diagnosis and poor outcomes. These children and their families should be provided with extra support throughout their course of treatment to increase successful treatment outcomes. Additionally, the type of facility a child is treated in may impact the course of their treatment completion and success. Facilities require investment into their staff and processes to diagnose and support patients throughout the course of their treatment.
